# Novel Role of Mitochondrial Manganese Superoxide Dismutase in STAT3 Dependent Pluripotency of Mouse Embryonic Stem Cells

**DOI:** 10.1038/srep09516

**Published:** 2015-03-30

**Authors:** Preethi Sheshadri, Ashwathnarayan Ashwini, Sowmya Jahnavi, Ramesh Bhonde, Jyothi Prasanna, Anujith Kumar

**Affiliations:** 1 School of Regenerative Medicine, Manipal University, Bangalore-560065

## Abstract

Leukemia Inhibitory Factor (LIF)/Signal transducer and activator of transcription 3 (STAT3) signaling pathway maintains the stemness and pluripotency of mouse embryonic stem cells (mESCs). Detailed knowledge on key intermediates in this pathway as well as any parallel pathways is largely missing. We initiated our study by investigating the effect of small molecule Curcumin on various signalling pathways essential for self-renewal. Curcumin sustained the LIF independent self-renewal of mESCs and induced pluripotent stem cells (miPSCs) in a STAT3 activity dependent manner. Gene expression analysis showed LIF/STAT3 and redox signaling components to be majorly modulated. Amongst ROS genes, expression of Manganese Superoxide Dismutase (MnSOD) specifically relied on STAT3 signaling as evidenced by STAT3 inhibition and reporter assay. The silencing of MnSOD, but not Cu-ZnSOD expression, resulted in the loss of mESC pluripotency in presence of LIF, and the overexpression of MnSOD is sufficient for maintaining the expression of pluripotent genes in the absence of STAT3 signaling. Finally, we demonstrate MnSOD to stabilize the turnover of pluripotent proteins at the post-translational level by modulating proteasomal activity. In conclusion, our findings unravel a novel role of STAT3 mediated MnSOD in the self-renewal of mESCs.

ESCs are unique cells with quintessential properties of self-renewal, maintaining proliferation without karyotypic abnormalities, and ability to make all the somatic cell types both *in vitro* and *in vivo*^1^,^2^. The unique self-renewal state of embryonic stem cells relies on the combinatorial expression of core transcription factors *Oct4*, *Sox2* and *Nanog*^3^,^4^ maintained by decisive instructions from exogenous and endogenous growth factors^5^,^6^. The classic methods for maintenance of the cardinal properties of undifferentiated phenotype and pluripotency of mESCs is to co-culture with inactivated mouse embryonic fibroblasts (MEF) feeder along with supplementation of medium containing leukemia inhibitory factor (LIF)^7^. LIF binds to class I cytokine receptors, LIF receptor (LIF-R) and gp130 leading to phosphorylation and activation of associated JAK tyrosine kinases. Consequence of this activation leads to Phosphorylation of latent transcription factor STAT3 and subsequent translocation into the nucleus to activate genes that are necessary for maintaining the ES cell pluripotency and self- renewal^8^,^9^. Several downstream targets of STAT3 includes Kruppel-like factor 4 (KLF4)^10^, FOXM1^11^, Tfcp2L1^12^ and GBX2^13^ that are shown to promote ESC self-renewal. However, LIF mediated STAT3 activation alone is insufficient to sustain the self-renewal property of ESCs and does in conjunction with BMP4 or fetal bovine serum^14^. Apart from the linear LIF/STAT3 pathway, a parallel PI3 kinase/Akt pathway emanating from LIF/gp130 based inputs has also been implicated in the maintenance of core pluripotent circuitry and self-renewal^15^. It is unlikely that a complex state of pluripotency is defined by such minimal signaling instructions from few pathways. Exploring the growth factors, novel small molecules and different pathways that promote ESC pluripotency and self-renewal provides the paradigm for understanding the role of different proteins involved in the self-renewal of stem cells, which is of great interest to the field of stem cell biology and regenerative medicine.

Recent studies have unravelled the role of different small molecules in maintenance of pluripotency in ESCs^16^,^17^. Curcumin, a diferuloylmethane compound, is one such small molecule that is found to modulate several signaling pathways and enzymatic activities like MAPKs, a serine/threonine-specific protein kinases^18^, EGF-induced phosphorylation of ERK1/2^19^,^20^,^21^, nitric oxide synthase activity, NF-κB activity, protein kinase C activity and production of reactive oxygen species (ROS)^20^,^22^. Many of the signaling pathways mentioned above have been reported to play key role in maintaining self-renewal or differentiation of ESCs^23^. However, it is unclear about the role of Curcumin in self-renewal or differentiation of mESCs and the signalling pathways adopted by this small molecule in exerting its effect. To address this question we evaluated the efficacy of Curcumin to maintain mESC cultures in LIF and feeder free culture conditions. By this approach and in combination with gene expression profiling, we demonstrate Curcumin to impart its effect on self-renewal of mESCs by modulating STAT3 mediated MnSOD expression. Further we provide experimental evidence in support of the essential role of MnSOD, but not CuZnSOD, in the self-renewal of mESCs. Elucidation of these unexplored proteins in ESC self-renewal provides newer insights for genes and signaling pathways that would facilitate better practical applications of ESCs.

## Results

### Curcumin sustains the pluripotency and self-renewal of mouse pluripotent cells in absence of LIF and Feeder

**Figure 1:**
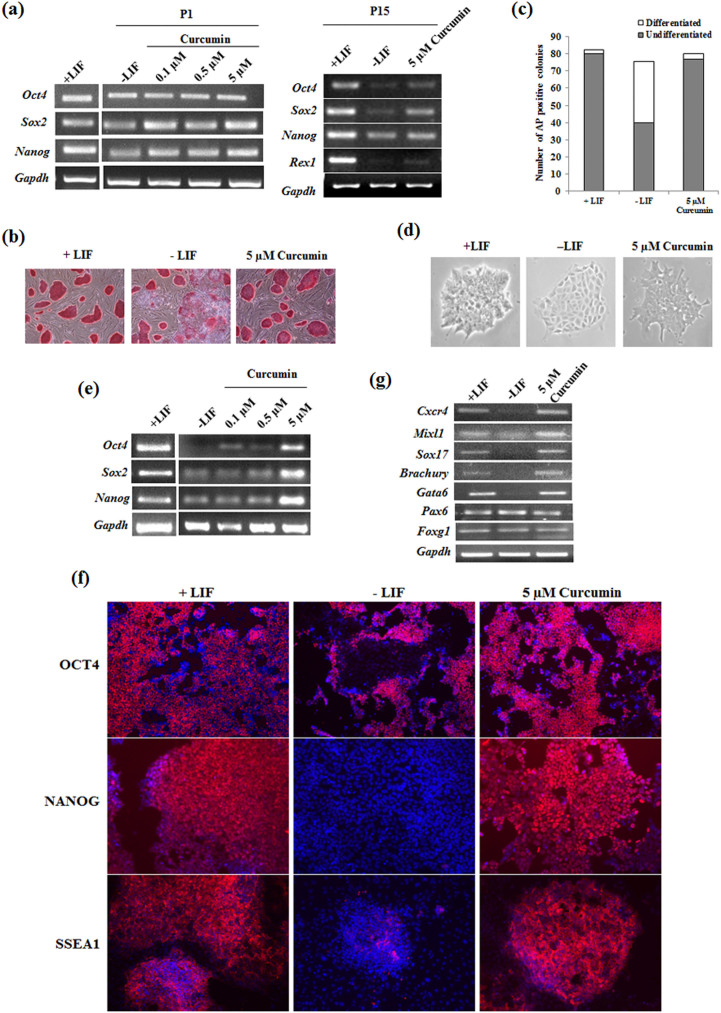
Curcumin sustains pluripotency of mESCs in the absence of LIF and feeder. (a) Cropped gel shows the pluripotent gene expression analysis of mESCs cultured in absence of LIF and in presence of different concentrations of curcumin at passage 1 and passage 15. Alkaline phosphatase staining of mESCs treated with curcumin in the absence of LIF (b) and graph represents (c) the manual counting of the number of differentiated and undifferentiated colonies. (d) Bright field images of mESCs cultured in the absence of LIF and feeders and in the presence of 5 µM Curcumin. Magnification – 10X; Nikon Eclipse TE-2000-S (e) Cropped gel shows the transcript analysis of pluripotent genes of mESCs treated with different concentration of Curcumin and cultured in the absence of LIF and feeders. (f) Immunofluorescent images of pluripotent proteins of mESCs treated with Curcumin and cultured in the presence or absence of LIF. Scale – 10 µm. (g) Expression analysis of the representative germ layer genes from the EBs obtained from the cells cultured in presence or absence of LIF and curcumin till passage 11.

To find the effect of Curcumin on self-renewal of ESCs, we cultured R1 mESCs on inactivated MEF in the absence of LIF and in the presence of various concentration of Curcumin along with DMSO as the solvent control. Although at passage 1 there was no significant difference between Curcumin treated and untreated cells, during later passages (15^th^ passage), ESCs cultured in absence of LIF lost the expression of pluripotent genes and cells cultured without LIF but in presence of 5 μM Curcumin significantly maintained the expression of pluripotent genes similar to that of mESCs cultured in presence of LIF (Fig. 1a, Supplementary Fig. 1d). Quantitative measurement of Curcumin's effect on pluripotency by alkaline phosphatase staining showed 76% ± 3.5 of Curcumin treated cells to be undifferentiated as compared to 40% ± 4.1 and 80% ± 2 of cells cultured in the absence and presence of LIF respectively (Fig. 1b, 1c). Previous reports had shown MEF to secrete LIF and support the self-renewal of mESCs^24^. To rule out the contribution of feeders in the expression of pluripotent genes, mESCs were cultured in the presence of Curcumin and in the absence of LIF and feeders. 5 µM Curcumin treated cells maintained the compact morphology of mESCs similar to the LIF treated cells (Fig. 1d). Curcumin treated cells in absence of LIF and feeder maintained the expression of *Oct4, Sox2* and *Nanog* at the transcript (Fig. 1e) and protein levels (Fig. 1f) similar to that of mESCs cultured in the presence of LIF. Even at the clonal level, alkaline phosphatase staining showed 49.8% ± 2.33 of Curcumin treated mESCs to be undifferentiated compared to 60.3% ± 4.38 and 1.45% ± 0.86 in presence or absence of LIF respectively (Supplementary Fig. 1b, 1c). The pluripotency at clonal level was further validated by SSEA1 staining (Supplementary Fig. 1a). To affirm the pluripotent property of Curcumin treated mESCs, we subjected these cells for *in vitro* and *in vivo* differentiation ability by embryoid body and teratoma formation respectively. Curcumin or LIF treated EBs expressed representative genes of all the three germ lineages. However, cells cultured without LIF and subjected to EB formation were found to be restricted only to the expression of ectodermal genes *Pax6* and *Foxg1* (Fig. 1g). It was also intriguing to know whether Curcumin had the ability to restrain further differentiation once it has been initiated. Addition of curcumin post EB formation did not restrict the expression of representative genes of any germ layers and on the other hand enhanced the expression of mesendodermal genes compared to untreated EBs (Supplementary Fig. 2). This data conveys that curcumin encourages the formation of mesendoderm lineage once the differentiating event has been commenced and by itself fails to assist the reprogramming event of the differentiated cells by inhibiting any of the three lineages. The pluripotency of Curcumin treated ES cells without LIF and feeders were further confirmed by teratoma formation, which contained the derivatives of all the three germ layers (Supplementary Fig. 3). These data demonstrate the ability of Curcumin to sustain the pluripotency of mESCs in absence of LIF and feeder.

**Figure 2:**
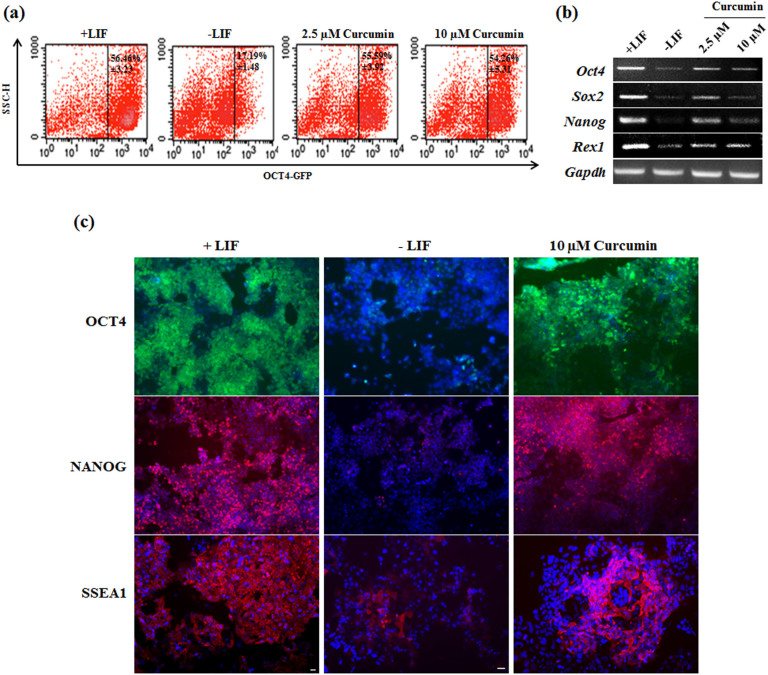
Curcumin sustains self-renewal of iPSCs in absence of LIF. (a) Flow Cytometric analysis of GFP expression in Oct4-GFP iPSCs cultured for 4–5 days in the absence of LIF and presence or absence of Curcumin. Data is representative of three independent experiments. Graph is represented as mean ± S.E.M. (b) Pluripotent gene expression analysis of Oct4-GFP iPSCs treated with Curcumin and cultured in presence or absence of LIF. (c) Immunofluorescence of Oct4-GFP iPSCs for pluripotent protein gene expression. Expression analysis of OCT4 was indirectly analyzed by measuring the expression of GFP as it is under the control of Oct4 promoter. Scale – 10 µm.

To confirm the role of Curcumin in sustenance of pluripotency in an independent pluripotent stem cell line, we chose Oct4–GFP transgenic induced pluripotent stem cell (iPSC) line wherein expression of GFP is under the control of *Oct4* promoter. Oct4-GFP cells lost GFP expression in 4–6 days in the absence of feeder cells and LIF, indicating a robust assay system for evaluating self-renewal. mESCs cultured in absence of LIF and feeder cells and in the presence or absence of different concentrations of Curcumin for 4 days were analyzed for the expression of GFP by FACS. mESCs exposed to Curcumin showed enhanced expression of GFP (55% ± 3.9) compared to cells cultured in the absence of Curcumin (17% ± 1.4) (with LIF as a positive control showing 56% ± 3.2 GFP expression) (Fig. 2a). Curcumin treated iPSCs maintained the expression of pluripotent genes at transcript and protein level similar to LIF treated mESCs (Fig. 2b, 2c). Expression of OCT4 was indirectly analyzed by the GFP expression as GFP ORF is in tandem with *Oct4* promoter (Fig. 2c). These results confirmed the role of Curcumin in sustaining the self-renewal of iPSCs, an independent pluripotent line.

### Curcumin maintains self-renewal of mESCs despite expression of Cyclin dependent kinase (CDK) inhibitor

**Figure 3:**
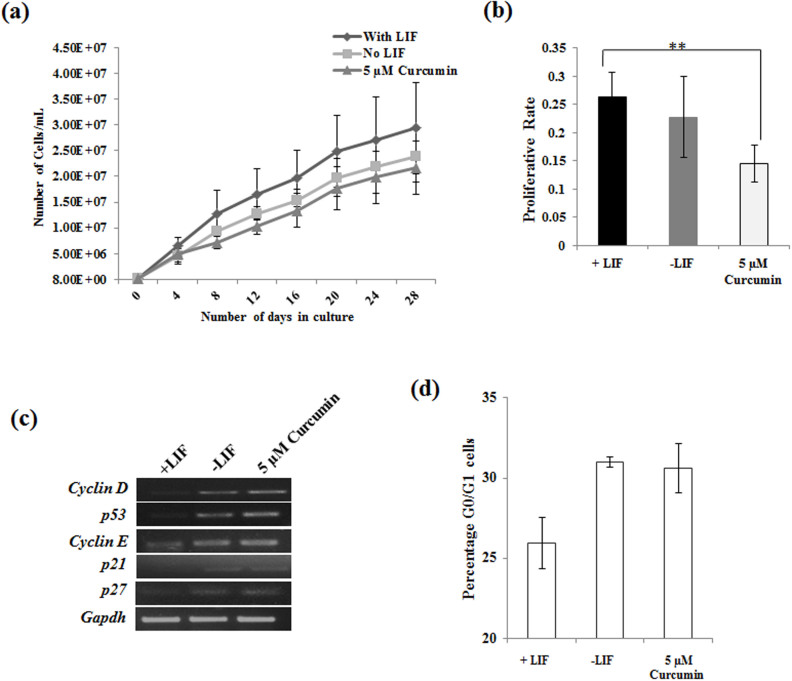
Curcumin maintains the self-renewal of mESCs independent of its proliferative status. (a) Cell proliferative assay of mESCs treated in the presence and absence of curcumin by trypan blue exclusion method. mESCs cultured in presence and absence of Curcumin for 11 passages with a starting cell number of 80,000 cells and cumulative population doubling calculated. Data is representation of experiments conducted in triplicates, mean ± S.E.M. (b) Cell proliferation analyzed by BrDU incorporation in mESCs treated with Curcumin in absence of LIF. (n = average of 3 independent experiments, mean experiments ± S.E.M, **, *p*<0.001). (c) Expression analysis of cell cycle proteins in curcumin treated mESCs and compared with mESCs cultured in presence or absence of LIF. (c) Cell cycle analysis of LIF deprived mESCs treated with Curcumin and stained with PI.

To understand the mechanism by how curcumin executes its function and as cell cycle regulation is one of the intertwined mechanism underlying pluripotency, we decided to analyze the cell cycle aspect of Curcumin treated mESCs. Cumulative population doubling of curcumin treated cells passaged through 8 passages showed significant decrease in population doubling compared to that of LIF treated cells (Fig. 3a). Analysis of proliferation rate by BrDu incorporation showed decreased proliferation of Curcumin treated cells compared to LIF treated cells (Fig. 3b). Transcript analysis of genes involved in the cell cycle showed enhanced expression of Cyclin D and cell cycle inhibitory genes *p21cip1*, *p27kip1* and *p53* in without LIF and Curcumin treated cells as compared to that of cells treated with LIF (Fig. 3c). Cell cycle analysis demonstrated larger population of Curcumin treated cells to be in G0/G1 phase, similar to that of differentiated mESCs cultured in LIF deprived condition (Fig. 3d). Although, Curcumin maintains the expression of pluripotent genes in absence of LIF, the above data demonstrates that their expression of cell cycle genes is similar to differentiated cells.

### Curcumin majorly modulates redox pathway genes

**Figure 4:**
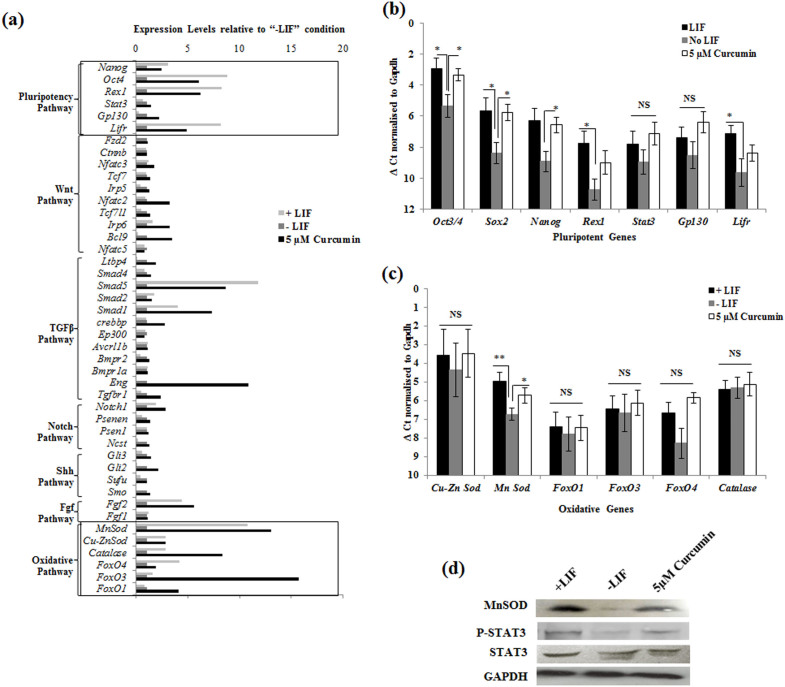
Modulation of different ES regulatory pathways by curcumin. (a) mESCs were cultured till confluency in the presence or absence of LIF and in presence of 5 µM Curcumin and analyzed for the expression levels of various genes belonging to different pathways involved in maintenance of pluripotency in mESCs. The expression levels were densitometrically analyzed and normalized with *Gapdh* expression and the values are plotted as relative expression levels in comparison with cells cultured in the absence of LIF. (b) qPCR analysis of pluripotent genes and genes involved in LIF/STAT3 pathway of mESCs treated with Curcumin. Data represented as mean ± S.E.M of 3 sets of experiments, *−*p*<0.05. (c) qPCR analysis of genes involved in oxidative pathway of cells treated with Curcumin. Data represented as mean ± S.E.M of 3 sets of experiments, *− *p*<0.05; **− *p*<0.001. (d) Western analysis of MnSOD, STAT3 and pSTAT3 in Curcumin treated and cells cultured in presence or absence of LIF conditions.

As cell cycle data was similar to LIF deprived ESCs and did not reveal the mechanism of Curcumin's pluripotent effect, we investigated the differential expression of genes of several pathways that are involved in the maintenance of pluripotency in mESCs. We performed transcript array of various genes involved in LIF/STAT3, FGF, NOTCH, TGFβ, SHH, WNT and oxidative pathways by RT-PCR (Supplementary Fig. 4a–e). Array data suggested that although Curcumin regulated the expression of few genes in the set of each signaling pathways involved in pluripotency, the LIF/STAT3 and oxidative pathways were majorly modulated (Fig. 4a). Quantitative assessment of pluripotent and redox genes by qRT-PCR confirmed the results obtained from RT-PCR (Fig. 4b, 4c). Further, Western analysis showed similar enhanced expression of MnSOD and pSTAT3 in LIF treated and curcumin treated cells compared to LIF deprived cells (Fig. 4d). These results demonstrated the probable role of oxidative pathway proteins in Curcumin mediated self –renewal of mESCs.

### MnSOD is a direct downstream target of the LIF/STAT3 pathway

**Figure 5:**
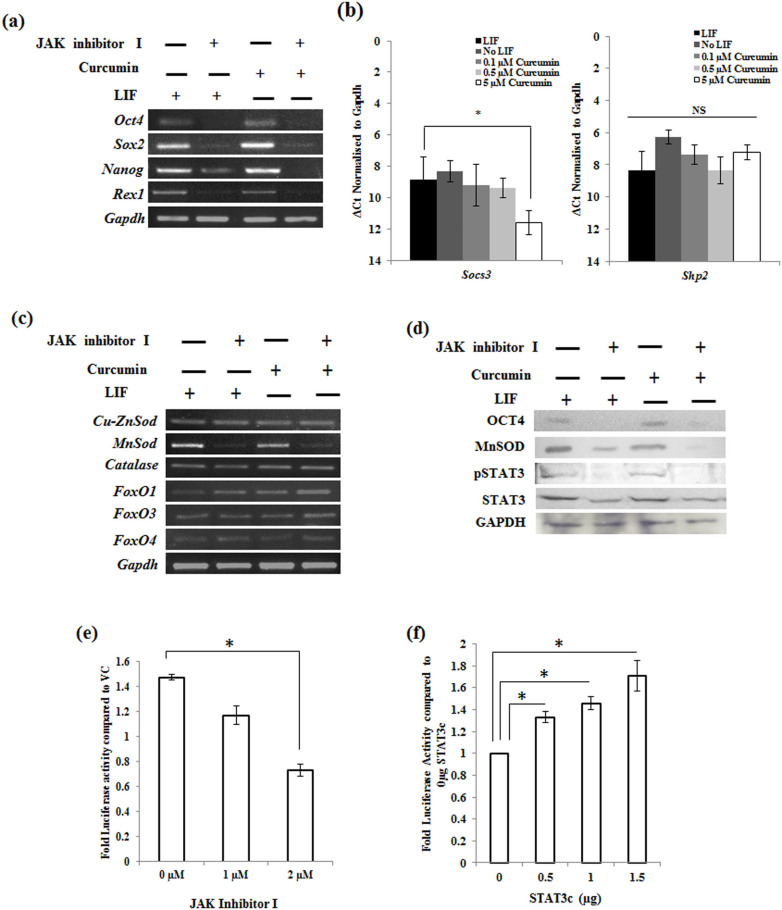
STAT3 plays a key role in Curcumin mediated pluripotency of mESCs. (a) mRNA expression analysis of pluripotent genes in feeder-free culture of Curcumin treated cells exposed to 2 µM JAK inhibitor I for 24 hrs. (b) Transcript analysis of STAT3 inhibitor genes in cells treated with different concentrations of Curcumin. Data represented as mean ± S.E.M of 3 sets of experiments, *- *p*<0.05. (c) Transcript analysis of redox pathway genes in mESCs treated with curcumin in the presence and absence of 2 µM JAK inhibitor I. (d) Western blot analysis of OCT4, STAT3, pSTAT3 and MnSOD in cells cultured in presence or absence of LIF, Curcumin and JAK inhibitor I. GAPDH was used as a protein loading control (e) The response of MnSOD promoter to LIF signaling was measured by treating mESCs with different concentration of JAK inhibitor I using Luciferase reporter assay. Data represented as mean ± S.E.M of 3 sets of experiments, * denotes *p* ≤ 0.05. (f) The interaction between STAT3 and MnSOD promoter determined by transfecting different concentrations of pMXs-STAT3 in 293T cells harboring MnSOD Luciferase reporter construct and measuring luciferase expression levels. Data represented as mean ± S.E.M of 3 sets of experiments, * denotes *p* ≤ 0.05.

The LIF/STAT3 pathway is the prominent and well elucidated pathway in the maintenance of pluripotency of mESCs^11^. As LIF/STAT3 pathway is also majorly modulated by Curcumin, we were curious to know whether STAT3 pathway is involved in the Curcumin mediated self-renewal of mESCs. We pretreated mESCs with Curcumin and subsequently treated with 2 µM JAK inhibitor I for 24 hrs. The cells treated with JAK inhibitor I appeared differentiated and showed a decreased expression of pluripotent genes. However, inhibition of STAT3 pathway also resulted in decreased expression of pluripotent genes in the Curcumin treated cells and abrogated the self-renewal property of Curcumin (Fig. 5a). This information suggested that the Curcumin mediated self-renewal of mESCs is *via* STAT3 pathway. To understand by what means curcumin maintains the pSTAT3 level in mESCs, we analyzed the expression of genes *Socs* (Suppressors of cytokine signaling) 3 and *Shp* (Protein phosphatase) 2, the proteins known to negatively regulate the JAK/STAT pathway. With increasing concentration of curcumin we found significant decrease in the expression of *Socs3* denoting the probable mechanism underlying curcumin mediated maintenance of pSTAT3 level (Figure 5b).

As the genes of LIF/STAT3 and oxidative pathway were majorly modulated in Curcumin treated cells, we were interested in knowing whether there exists a “linear correlation” between these two pathways. Among the oxidative genes analyzed in the JAK inhibitor I treated cells, expression of *MnSod* was specifically down modulated in the absence of STAT3 signaling (Fig. 5c). Modulation of *MnSod* gene expression by Curcumin was further confirmed by demonstrating the increased expression of *MnSod* with increasing concentration of Curcumin (Supplementary Fig. 4f). Western analysis confirmed the above observation at the protein level (Fig. 5d). In addition, *MnSOD* promoter activity was induced when constitutive STAT3 and *MnSOD* promoter-Luciferase construct was transfected in 293T cells (Fig. 5f). Similarly, downregulation of *MnSOD* promoter activity upon addition of JAK inhibitor in mESCs provided direct evidence that the *MnSod* induction by STAT3 happened at its transcriptional level (Fig. 5e). A literature survey showed MnSOD to harbor putative STAT3 binding site on the promoter region and thus acting as a direct downstream target of STAT3 in neuronal cells^25^. Together these results suggested Curcumin's effect of maintaining mESC pluripotency is via STAT3 signaling and that the transcription of *MnSOD* stimulated by LIF and curcumin is directly regulated by STAT3.

### MnSOD is essential and sufficient to maintain the pluripotency of mESCs in the absence of LIF

**Figure 6:**
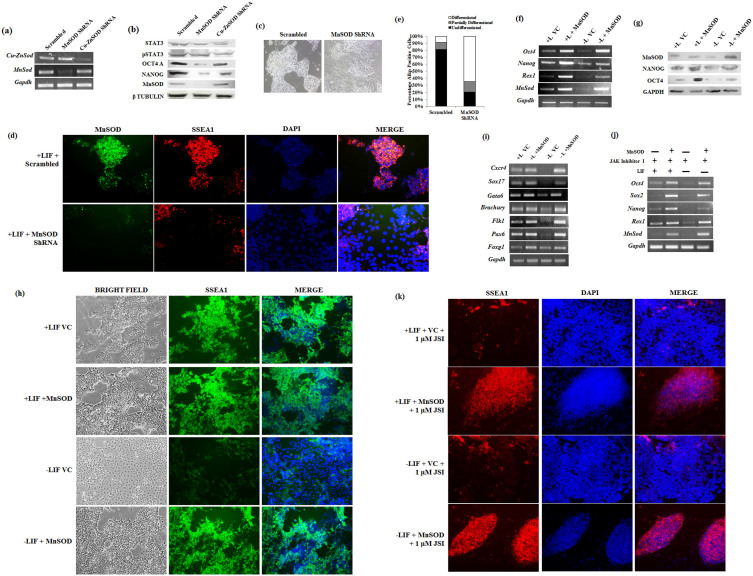
MnSOD is essential and sufficient to maintain the expression of pluripotent genes. (a) RT-PCR analysis of *MnSod* and *Cu-ZnSod* in mESCs transfected with ShRNAs specific to *MnSod* and *Cu-ZnSod*. (b) Western blot analysis of STAT3, pSTAT3, OCT4, NANOG and MnSOD proteins in mESCs transduced with ShRNAs specific to *MnSod* and *Cu-ZnSod*. (c) Bright field image of mESCs lentivirally transduced with Scrambled or MnSOD ShRNA. Magnification – 10X; Nikon Eclipse TE-2000-S. (d) Immuno-fluorescence images of mESCs transduced with scrambled or shRNA specific for MnSOD and cultured for 4 passages. Magnification – 20X. (e) Manual enumeration of the number of differentiated, undifferentiated and partially differentiated mESC colonies transduced with scrambled or MnSOD ShRNA, as observed by alkaline phosphatase staining at passage 4. Data represented as mean of n = 3 experiments. (f) The effect of MnSOD overexpression on the transcript levels of pluripotency genes in mESCs cultured in presence or absence of LIF. (g) Western blot analysis of pluripotent proteins in mESCs overexpressing MnSOD and respective vector controls. (h) Bright field and SSEA1 staining images of mESC cultured either in presence or absence of LIF and with or without MnSOD overexpression. Scale – 10 µm. (i) Transcript analysis of representative genes of three germ layers of EB differentiated mESCs transfected with MnSOD or vector control and cultured in presence or absence of LIF. (j) Transcript analysis of pluripotent genes in mESCs transiently overexpressing MnSOD and treated with 1 µM JAK inhibitor I for 48 hrs. (k) Immunofluorescence imaging of SSEA1 protein expression in mESCs retrovirally transduced with MnSOD and vector control and subsequently treated with 1 µM JAK inhibitor I for 4 passages. Magnification – 20X.

Among the oxidative genes analyzed, as MnSOD is specifically modulated by STAT3 signaling pathway, we were interested in knowing the role of MnSOD in pluripotency and self-renewal of mESCs. Treating mESCs with increasing concentration of MnSOD specific inhibitor, 2-Methoxyoestradiol (2-MeOE2)^26^ showed decreased SOD activity (Supplementary Fig. 5a). Simultaneously, there was significant decrease in the expression of pluripotent genes in the cells exposed to 2-MeOE2 and treated with LIF or Curcumin (Supplementary Fig. 5b). Specific inhibition of *MnSod* by 2-MeOE2 is demonstrated by unaltered expression of *Cu-ZnSod* in presence or absence of 2-MeOE2. Surprisingly, despite the presence of *CuZnSod* in the cells exposed to 2-MeOE2 and LIF, *Cu-ZnSod* failed to sustain the expression of pluripotent genes and thereby reiterated the specific role of MnSOD in pluripotency of mESCs (Supplementary Fig. 5b). To specifically signify the essential role of MnSOD, shRNA mediated downregulation of *MnSod* and *Cu-ZnSod* were generated in mESCs cultured in presence of LIF (Fig. 6a). Down regulation of MnSOD, resulted in the loss of typical mESC morphology at passage 4 (Fig. 6c) and lost pluripotency as depicted by lack in alkaline phosphatase staining (Fig. 6e). Colocalization study further showed a dramatic decrease of pluripotent gene SSEA1 in the cells lacking MnSOD (Fig. 6d). In addition, MnSOD lacking mESCs, but not CuZnSOD, showed decreased expression of pluripotent proteins without affecting the STAT3 phosphorylation levels (Fig. 6b). This suggested MnSOD to be the downstream player of STAT3 and abrogation of MnSOD depletes the pluripotency of mESCs despite presence of active STAT3 signaling. On the other hand, down regulation of Cu-ZnSOD expression did not alter the expression of pluripotent proteins, suggesting the essential and specific role of MnSOD in maintenance of pluripotency of mESCs.

To conclusively demonstrate the role of MnSOD in the pluripotency of mESCs, we overexpressed MnSOD in mESCs cultured in presence or absence of LIF and further analyzed the pluripotent gene expression. As consistent with previous results, cells cultured in the absence of LIF and feeders showed decreased expression of pluripotent genes as compared to cells exposed to LIF. Surprisingly, cells overexpressing MnSOD and cultured in absence of LIF maintained higher expression of pluripotent genes at the transcript and protein level in contrast to cells transfected with vector control (Fig. 6f, 6g). We also found that the morphology and SSEA1 expression of MnSOD transfected mESCs in the absence of LIF and feeder layers is indistinguishable from that of mESCs cultured on LIF (Fig. 6h, Supplementary Fig. 6c). Quantitative analysis further showed 75% ± 0.03 of mESCs deprived of LIF and overexpressing MnSOD to be SSEA1 positive compared to 52 ± 2.11 of mESCs transfected with vector control (Supplementary Fig. 6c). To test the efficiency of passagability, we obtained stable expression of MnSOD by transducing a retroviral construct carrying MnSOD. Compared to vector control, MnSOD overexpressing mESCs maintained the expression of pluripotent genes beyond passage 10 in LIF deprived condition (Supplementary Fig. 6d). Even at the clonal level, alkaline phosphatase staining of mESCs transduced with MnSOD and cultured in absence of LIF was comparable with that of mESCs cultured in presence of LIF (Supplementary Fig. 6a, 6b). Further, embryoid body differentiation to all the three germ layers by MnSOD overexpressing mESCs, in contrast to LIF deprived vector control cells, proved the pluripotency of MnSOD overexpressing mESCs in absence of LIF (Fig. 6i). To ascertain the effect solely to MnSOD and not due to contaminating STAT3 signaling, mESC overexpressing MnSOD and cultured in presence or absence of LIF were treated with JAK inhibitor I. Despite inhibition of STAT3 pathway, mESCs overexpressing MnSOD maintained the expression of pluripotent genes at transcript and protein level in contrast to LIF deprived vector control cells (Fig. 6j). Furthermore, mESCs harboring stable MnSOD expression maintained the expression of pluripotent protein SSEA1 up to passage 4 even after abolishing STAT3 signaling by JAK inhibitor I (Fig. 6k). These data conclusively demonstrated the ability of MnSOD to maintain the expression of pluripotent genes in the absence of LIF.

### MnSOD augments the stability of pluripotent proteins by modulating proteasome activity

**Figure 7:**
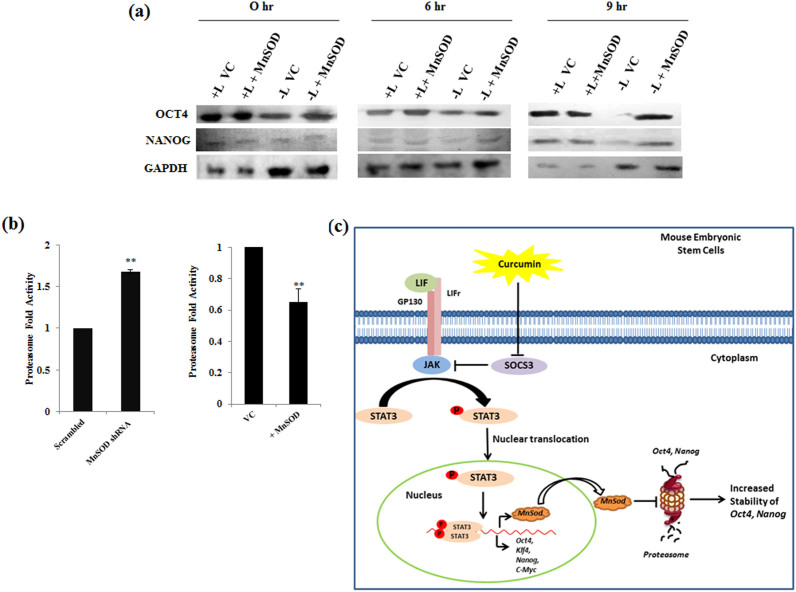
MnSOD maintains pluripotency by modulating protein stability and proteasome activity. (a) Stability of pluripotent proteins OCT4 and NANOG were measured by Western blot analysis of mESCs transfected with either vector control or MnSOD and cultured in presence or absence of LIF and treated with 50 ug/ml cycloheximide for different time periods. (b) Proteasome activity of mESCs either overexpressing or depleted of MnSOD was measured using cytosolic extract and proteasome specific substrate Suc-LLVY-AMC. Y-axis represents the arbitrary values of proteasomal activity. Data represented as mean ± S.E.M of 3 sets of experiments, **, *p* ≤ 0.01. (c) Schematic representation of the novel pathway followed by MnSOD in maintaining pluripotency of mESCs. Curcumin, by downregulating SOCS3 enhances STAT3 mediated MnSOD expression and inturn MnSOD increases the stability of pluripotent proteins OCT4 and NANOG by decreasing proteasomal activity.

To identify the downstream mechanism of how MnSOD maintains the expression of pluripotent genes, we sought to analyze the stability of pluripotent proteins. We followed the degradation of pluripotent proteins OCT4 and NANOG during the blocking of nascent protein synthesis by cycloheximide. Post Cycloheximide treatment for 9 hrs. pluripotent proteins appeared stable in mESCs exposed to LIF compared to without LIF condition. Overexpression of MnSOD in LIF deprived condition rescued the stability of OCT4 protein similar to mESCs exposed to LIF (Fig. 7a). Majority of intracellular protein degradation is mediated by ubiquitin mediated proteasomal degradation pathway^27^. To have a closer look whether MnSOD modulates the proteasomal activity and thereby enhances the stability of pluripotent proteins, we checked the proteasomal activity in mESCs in presence and absence of MnSOD by using proteasome specific substrate Suc-LLVY-AMC. Silencing of MnSOD in mESCs enhanced the proteasomal activity compared to vector control and on the other hand there was nearly twofold reduction in proteasomal activity upon overexpression of MnSOD (Fig. 7B). These data demonstrates that the post translational stabilization of the pluripotent proteins by modulating proteasome mediated degradation is probably one of the underlying mechanisms by which MnSOD maintains pluripotency of mESCs.

## Discussion

Elucidation of different signaling events involved in the self-renewal of ESCs is important for understanding their physiology and optimizing culture conditions. A significant amount of research has been conducted on identifying the “switches” that dictate stem cells either to differentiate or to maintain pluripotency and self-renewal. Although, several studies have highlighted the dynamic changes in the ROS and antioxidant enzymes during the self-renewal and differentiation of ESCs, seclusion of prime antioxidant enzyme that plays the crucial role in the self-renewal of mESCs had not been achieved. In the present study we identified MnSOD as a critical LIF/STAT3 downstream target that mediates LIF/STAT3 self-renewal of mESCs (Fig. 7C). We also demonstrate MnSOD to execute its role by influencing the stability of pluripotent genes and modulating proteasomal activity.

Apart from LIF/STAT3 pathway, enormous effort has been put forth to unravel the various other conduits that could be involved in pluripotency of mESCs resulting in a LIF independent growth of mESCs. Small molecules like GSK-3 inhibitor CHIR99021 and MEK (MAPK/ERK kinases) inhibitors SU5402 and PDI184352 have provided great opportunity to gain new insights into alternative pathways that facilitates ES cell self-renewal^28^,^29^,^30^. Curcumin, one such small molecule, had not been tested previously for its role in maintenance of pluripotency. Curcumin is a well-known antioxidant, anti-inflammatory, antiviral, antimicrobial, antidiabetic and anticancer agent^31^,^32^. Although, there exists a previous report stating Curcumin to enhance the differentiation of human ESCs, the study was performed during the course of differentiation by EB method^33^. Similar experiment of adding Curcumin to mESCs during the course of EB formation did not restrain the cells from differentiation and ruled out the possibility of Curcumin maintaining the pluripotency by suppressing any of the derm layers. To understand by what means Curcumin maintains self-renewal of ESCs, we looked into two important facets of ESCs: a) Cell cycle parameters and b) different signalling pathways existing in ESCs. ESCs have unique cell cycle kinetics with very short G1 phase marked by decreased expression of CDK inhibitors and Retinoblatoma activity^34^. The present study evidenced Curcumin to maintain the self-renewal of mESCs despite decreased proliferation and simultaneous up regulation of cyclin-dependent kinase (CDK) inhibitor genes, a phenotype observed in mESCs deprived of LIF or in differentiated cells. In our study, when differentiation of mESCs was initiated by depriving LIF, there was increase in the expression of CDK inhibitors – p21, and p27 and a similar expression was maintained when cells were exposed to Curcumin without LIF. This denotes that Curcumin maintains the pluripotency of mESCs in absence of LIF and feeder without modulating the expression of CDK inhibitors. Recently, a similar report by Vazquez-Martin et al., showed small molecule Metformin to limit the proliferative capacity of iPSCs without compromising on their self-renewal property^35^. We thus reveal a novel characteristic property of Curcumin to maintain the stem cell properties independent of the proliferative capacity of the cells.

Screening for different signaling pathways known to play significant role in ESC pluripotency showed LIF/STAT3 and oxidative pathways to be majorly modulated by Curcumin. Further analysis demonstrated Curcumin to mediate its effect in a STAT3 signaling dependent manner. Previous reports had mentioned Curcumin, in a dose dependent manner to suppress STAT3 phosphorylation and concomitant decrease in proliferative capacity in cancer derived cells^36^. Although we observed a reduced proliferation of mESC exposed to curcumin, the suppression of STAT3 phosphorylation was not observed at the lower concentration used in our experiments. The JAK/STAT pathway is subject to negative regulation by protein family of phosphatases including SHPs, and PTP1B (protein tyrosine phosphatase 1b), PIAS (protein inhibitor of activated STAT) and SOCS proteins^37^. The present study in mESCs demonstrated curcumin to decrease the expression of *Socs3*, but not *Shp*2 and conveying the probable mechanism underlying the maintenance of pSTAT3 in absence of LIF. Conflicting reports exist in different cell types regarding Curcumin's role in modulating the SOCS3 expression. In hematopoietic progenitors obtained from myeloproliferative neoplasm patients, Curcumin elevated the transcript levels of *Socs1* and *Socs3* by inhibiting the histone deacetylase activity^38^. In fructose fed rats and obese mice, Curcumin has been shown to improve the leptin signaling in hepatic cells by increased JAK2 phosphorylation and downregulating SOCS3 expression^39^,^40^. Depending on the cell type, Curcumin exhibits contrapositive effects on STAT3 phosphorylation and SOCS3 expression”.

Our experimental results using JAK inhibitor I showed MnSOD, among different antioxidant genes analyzed, to be the downstream target of STAT3 involved in the sustenance of self-renewal of mESCs. MnSOD is the main antioxidant enzyme that scavenges ROS (specifically superoxide) in the inner mitochondrial matrix^41^. MnSOD is a nucleus encoded protein and has been demonstrated to act as a mitochondrial fidelity protein and beside its role in decreasing ROS levels, it is also involved in the mitochondrial biogenesis during differentiation of ESCs^42^. Pluripotent stem cells have reduced mitochondrial activity and undergo lesser oxidative stress^43^. During the course of differentiation, the oxidative pathway genes are modulated to maintain sufficient amount of ROS which is essential for the differentiation of ESCs^44^. Previous studies on stress defense in mESCs identified MnSOD to be one among group of genes down modulated during EB formation^45^. Similarly, gene profiling studies by Trouillas etal., with mRNAs from ES cells in presence and absence of LIF demonstrated down regulation of MnSOD with LIF withdrawal^46^. However, further studies dissecting the functional validation and relevance of MnSOD in pluripotency has not been observed. From the present and previous reports, it is convincing that despite decreased mitochondrial activity and mass in undifferentiated ESCs, these cells express increased amount of mitochondrial protein MnSOD compared to differentiated ESCs. Also, in our present report, not all the redox regulated genes like Catalase, CuZnSOD, FOXOs, etc. are regulated in a STAT3 dependent manner. Interestingly, mice with deletion of Cu-ZnSOD are viable whereas MnSOD-deficient (*Sod2*^-/-^) mice develop several pathologies and neonatal lethality (~100% mortality by day 10)^47^. All of this information argues for the specific modulation of MnSOD by STAT3 and emphasizes its precise role in ESC physiology.

To understand the intriguing puzzle of how MnSOD maintains the expression of pluripotent genes in the absence of LIF, we looked at the post translational stability of these proteins. Cycloheximide experiments demonstrated MnSOD to enhance the stability of the pluripotent proteins. Also, overexpression and repression studies clearly demonstrated MnSOD to supress proteasomal activity. These results signify a strong correlation between the stability of pluripotent proteins, proteasomal activity and MnSOD protein level. Previous reports have suggested that the homeostasis of pluripotent proteins including OCT4, NANOG and c-MYC to be regulated by ubiquitination^48^,^49^. Buckley etal., by using mass-spectrometry-based mapping demonstrated the distinct state of pluripotency to be mediated by ubiquitin proteasome system^50^. Hernebring etal., showed elevated activity of proteasome during differentiation of mESCs^51^, which very well corroborated with the silencing of MnSOD phenotype in the present study. Although our data cannot preclude the possibility of other mechanism, we comprehensively propose that MnSOD maintains the pluripotent state by decreasing the proteasome mediated degradation of pluripotency regulators. Hitherto, it was reported ROS to play a pivotal role in enticing ESCs towards differentiation and this is the first report unveiling the role of an active antioxidant component of redox pathway, MnSOD, in the self-renewal of mESCs.

Recently various necessitating transcription factors for pluripotency that are downstream target of STAT3 have been reported^11^,^46^. However, to the best of our knowledge, an indepth study of a STAT3 target which plays a pivotal role at the post translational level to sustain the self-renewal and pluripotency had not been performed. In this study, we report MnSOD, apart from maintaining the homeostasis of the cells by regulating ROS levels also plays a major role of maintaining pluripotency in mESCs. Our results herald a conceptual advance in the knowledge of stem cell signalling biology and identify MnSOD as a crucial intermediate downstream of STAT3 in facilitating the proper control of ESCs fate.

## Methods

### Cell Culture

mESCs(R1) and Oct4- GFP tagged miPSCs (Kind gift from Prof. Catherine Verfaillie, Leuven) were cultured on mitotically inactivated MEF feeders, in a suitable medium -DMEM-High Glucose (Gibco) containing 15% FBS (HiMedia), 2 mM Glutamine, 1X NEAA, 1% Penicillin and 1X Streptomycin (All from Gibco) along with 8 µM LIF (Chemicon). For inhibition of STAT3 or MnSOD, 10^5^ cells were plated onto gelatin coated 35 mm dishes and were induced with LIF (Millipore), No LIF and 5 µM Curcumin (Acros Organics) and 24 hrs post induction, the cells were treated with 2 µM Insolution JAK inhibitor I (Calbiochem) or 20 µM 2-Methoxyestradiol (Sigma Aldrich) respectively. 24 hrs post JAK inhibitor treatment, the cells were analyzed for gene and protein expressions while the 2-Methoxyestradiol treated cells were harvested 48 hrs post treatment. For details on clonal analysis, embryoid body differentiation, alkaline phosphatase assay, Teratoma formation, cumulative population doubling and BrDU based cell proliferation, see supplementary material.

### Cloning, Transfection, shRNA lentiviral production and Gene Expression Analysis

The Open Reading Frame (ORF) of MnSOD was PCR amplified from mESC cDNA using gene specific primers and cloned in BamHI and XhoI sites of pCDNA3.1 (+) (for transient transfection) and pMIG vector (for retroviral transfection) and positive clones were confirmed by sequencing (Amnion Biosciences). To clone MnSOD promoter region, 1779 bp upstream of MnSOD start site^25^ was PCR amplified and cloned into KpnI and XhoI regions of pGL3 basic vector (Addgene).

For transient transfection, 5 µg of plasmid was transfected into confluent mESCs using XtremeGENE HP transfection reagent (Roche Diagnostics) and 24 hrs post transfection, the transfected cells were subjected to different conditions (With LIF, No LIF) and were analysed for their gene and protein expression pattern. MnSOD overexpression was also performed by retroviral transduction using pMIG MnSOD and pMIG-GFP vector control. For knockdown of MnSOD and Cu-Zn SOD, the lentiviral vectors pLKO-MnSOD shRNA and pLKO-Cu-ZnSOD shRNA (Sigma Aldrich) along with the scrambled control were transduced in mESCs. For retroviral and lentiviral production and details on transduction see supplementary material.

RNA isolation was performed using RNeasy micro kit (Qiagen) as per manufacturer's instruction. For additional details on PCR, see supporting information Experimental Procedures. Primer sequences are described in supplementary table 1.

### Analysis of Protein

For immunofluorescence, cells were fixed in 4% paraformaldehyde, permeabilized and incubated with primary antibody at 4°C overnight, followed by incubation for 2 hrs at RT with Secondary antibody and incubated for 2 hrs at RT and observed under fluorescent microscope (Nikon Instruments Inc.). The primary and secondary antibodies and their dilutions used are described in Supplementary table 2.

For western blot, proteins were fractionated on 8–12% SDS-PAGE gels and transferred onto charged PVDF membrane (Millipore). Post transfer, the membrane was blocked using 3% Skimmed milk/BSA in 0.1% TBST solution and probed with primary antibody overnight at 4°C and subsequently with horseradish-peroxidase (HRP)-conjugated secondary antibody for 1 hr at RT. The primary antibodies, secondary antibodies and their dilutions are described in supplementary table 2. The protein specific bands were detected by chemiluminiscence analyser (GE ImageQuant LAS 4000,) or using a TMB substrate (Sigma Aldrich). For additional details, see supplementary material.

### Flow cytometric analysis

Enumeration of number of GFP positive cells or measuring SSEA1 postive cells stained with primary Anti-SSEA1 antibody (BD Biosciences, Catalogue number 557895) and PE conjugated secondary antibody (BD Biosciences, Catalogue number 349073) was performed using flow cytometer (BD FACSCalibur, BD Biosciences). For Propidium iodide staining, cells were stained with Propidium Iodide (Sigma Aldrich) and cell cycle was analysed by flow cytometry. For additional details, see supplementary material.

### Luciferase Assay

Cells were transfected with 950 ng of pGL3-MnSOD-luciferase and 50 ng of TK Renilla, along with vector control, using XtremeGENE HP transfection reagent. 24 hrs post transfection, the medium was replaced with fresh mESC medium and induced with different concentrations of JAK inhibitor I. 24 hrs post inhibitor treatment, the cells were harvested and luciferase activity was performed using dual luciferase reporter assay system (Promega Corporation) as per manufacturer's instructions.

To look at the direct interaction, 293T cells cultured in 24 well plates were transfected with 500 ng of pMIG-SOD2, 50 ng of TK Renilla and different concentrations of pMX-STAT3 (Addgene) along with vector control. 24 hrs post transfection, the cells were harvested and luciferase activity was performed using the dual luciferase activity kit (Promega) as per manufacturer's instructions.

### SOD activity

Cells were harvested 48 hr post treatment of different concentration of 2-methoxyestradiol and equal concentrations of protein were used for SOD activity analysis, using SOD activity kit as per manufacturer's instructions (Biovision).

### Proteasomal Activity

The cytosolic extract of MnSOD plasmid transfected and MnSOD ShRNA transduced mESCs were collected using lysis buffer^52^ and protein was estimated using BCA Protein Assay (Novagen) 5 µg of the cytosolic extracts was incubated with 100 µM Suc-LLVY-AMC substrate in Tris-HCl (pH-8.0) at 37°C for 2 hrs. Post incubation, the reaction was arrested using 100% ethanol and the absorbance was measured at 360–460 nm (Perkin elmer 1420 multilable plate reader).

### Statistical Analysis

Student's t-test was applied at appropriate places. The values with p ≤ 0.05 were considered statistically significant.

## Supplementary Material

Supplementary Information Supplementary Information
