# Supplementary material for: Novel Role of Mitochondrial Manganese Superoxide Dismutase in STAT3 Dependent Pluripotency of Mouse Embryonic Stem Cells
Source: Sci Rep. 2015 Mar 30;5:9516. doi: 10.1038/srep09516 (PMC5380158; doi:10.1038/srep09516)
Supplement: Supplementary Information [file srep09516-s1.pdf]

Supporting Information:

Novel Role of Mitochondrial Manganese Superoxide Dismutase in STAT3 Dependent Pluripotency of Mouse Embryonic Stem Cells

Preethi Sheshadri[1], Ashwathnarayan Ashwini[1], Sowmya Jahnavi[1], Ramesh Bhonde[1], Jyothi Prasanna[1], Anujith Kumar[1]

[1]School of Regenerative Medicine, Manipal University, Bangalore-560065

Running Title: Essential role of MnSOD in pluripotency of mESC

Key Words: Embryonic stem cells, Redox, Pluripotency, signaling, mitochondria, STAT3 pathway, Proteasome

*Corresponding author: Anujith Kumar, anujith.kumar@manipal.edu, Phone (+91) 80 28460671, FAX (+91)8028460672

Present address: School of Regenerative Medicine, Allalsandra, GKVK post, Yelahanka, Bangalore-560065, India

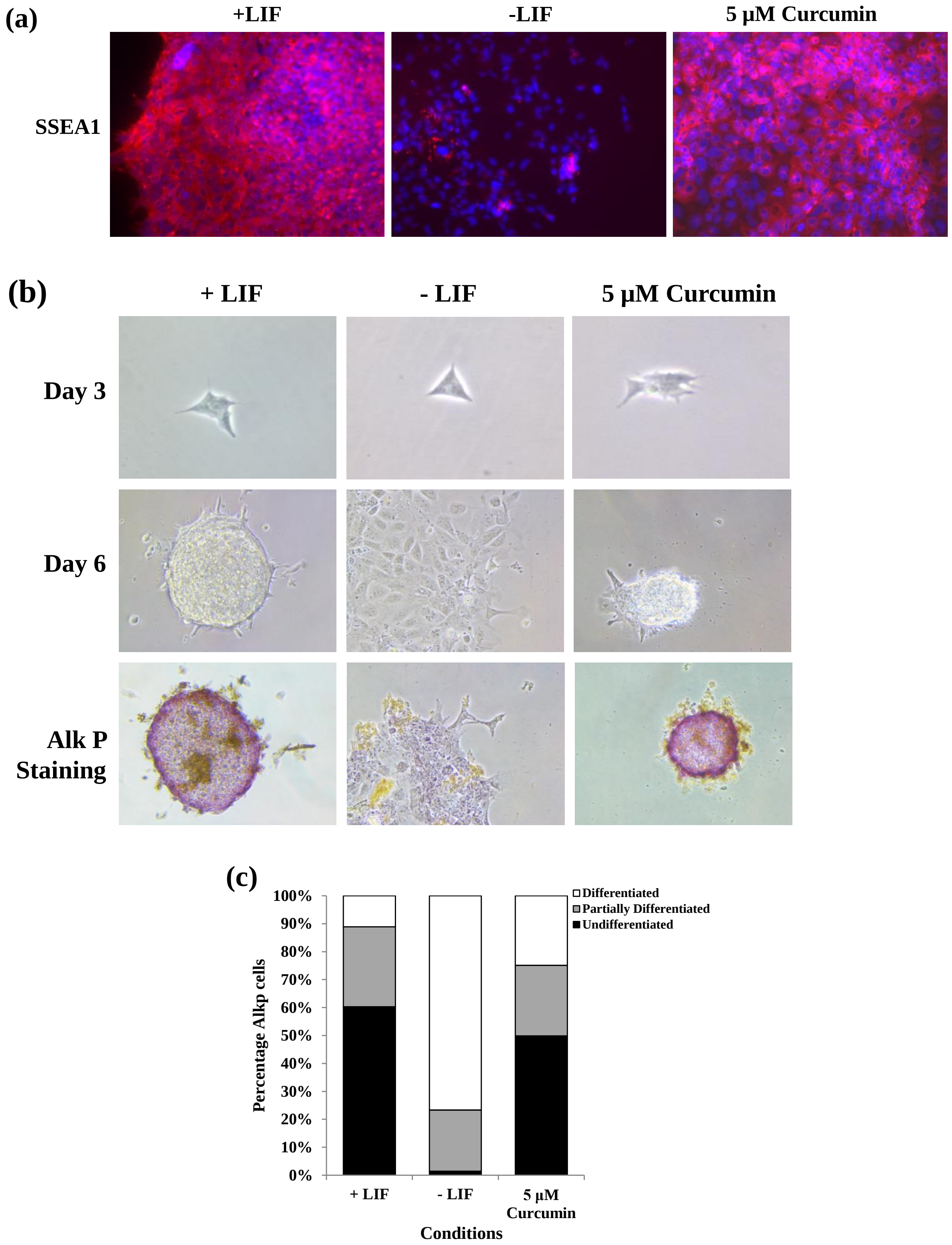
(a)
+LIF
-LIF
5 µM Curcumin
SSEA1
(b)
+ LIF
- LIF
5 µM Curcumin
Day 3
Day 6
Alk P Staining
(c)
100%
90%
80%
70%
60%
50%
40%
30%
20%
10%
0%
Percentage Alkp cells
Differentiated
Partially Differentiated
Undifferentiated
+ LIF
- LIF
5 µM Curcumin
Conditions

(d)

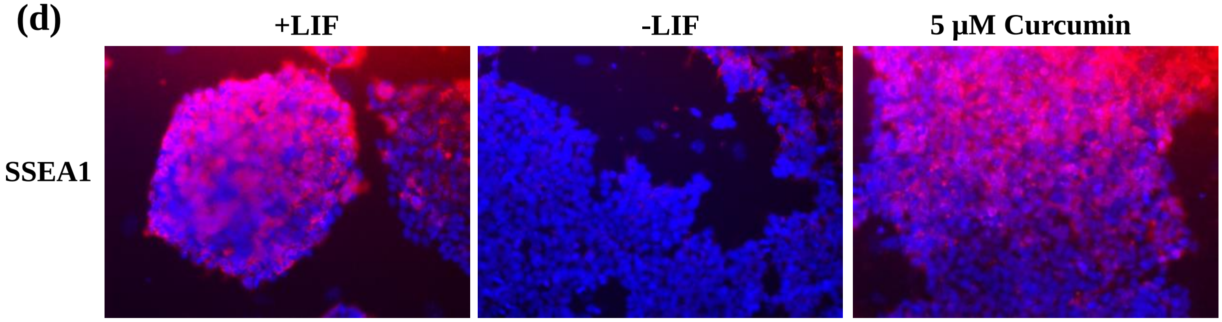

Supplementary Figure 1: Curcumin maintains the mESCs morphology in absence of LIF at clonal level. Approximately 10 cells were plated in each well of 12 well plate and cultured in the presence and absence of LIF and in the presence of 5 µM curcumin. The cells were tracked from day 3 till day 6. On day 6, immunofluorescence (a) and alkaline phosphatase staining (b) was performed and the number of alkaline phosphatase positive colonies were enumerated (c). Graph represents mean of 3 sets of experiments. (d) SSEA1 staining of mESCs cultured in the presence of feeders and in the presence and absence of LIF and curcumin for 14 passages by immunofluorescence. 20X magnified Phase Contrast Images and Alkaline Phosphatase stained images.

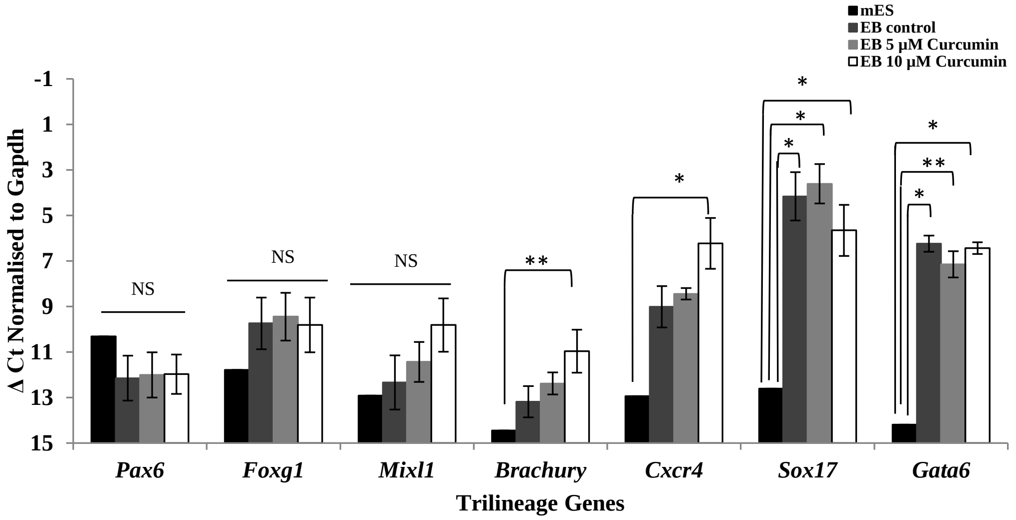

Supplementary Figure 2: Higher concentration of Curcumin drives the cells towards endodermal lineage. mESCs were cultured for 14 days in low adherent plates and treated with different concentrations of curcumin and in the absence of LIF. On day 14, the cells were harvested and gene expression analysis was performed by quantitative Real time PCR. Data represented as mean ± S.E.M of 3 sets of experiments, * - $p<0.05$; ** - $p<0.001$.

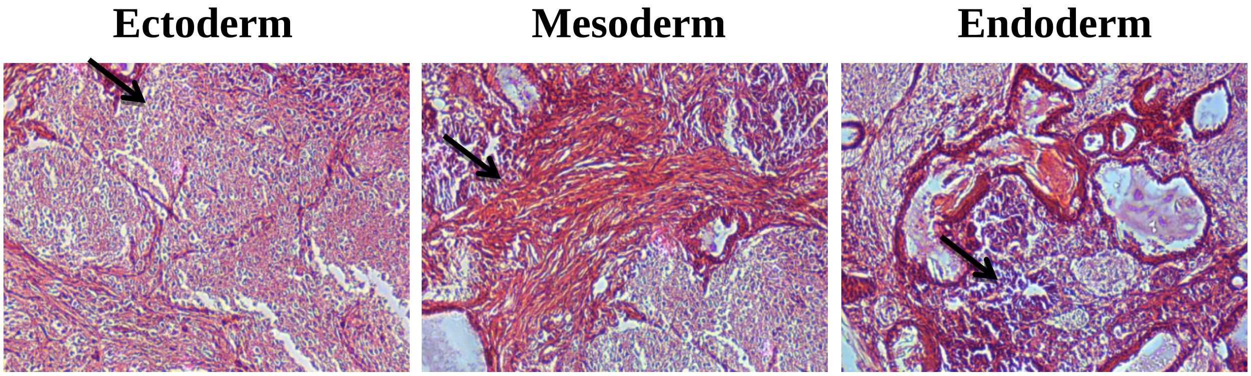

Supplementary Figure 3: Curcumin treated cells differentiate into all the three germ layers *in vivo*. Approximately 1-3*10^6 5 µM curcumin treated mESCs cultured in absence of LIF were injected into injected subcutaneously (s.c.) into the dorsal flank of 4-5 week old female athymic nude mice and maintained for 4 weeks post which the mice were sacrificed and H&E staining was performed on the teratoma tissue. The images are 10X magnified phase contrast images with the arrows indicating – glial ectoderm cells, cartilaginous mesodermal cells and lumen like endodermal cells.

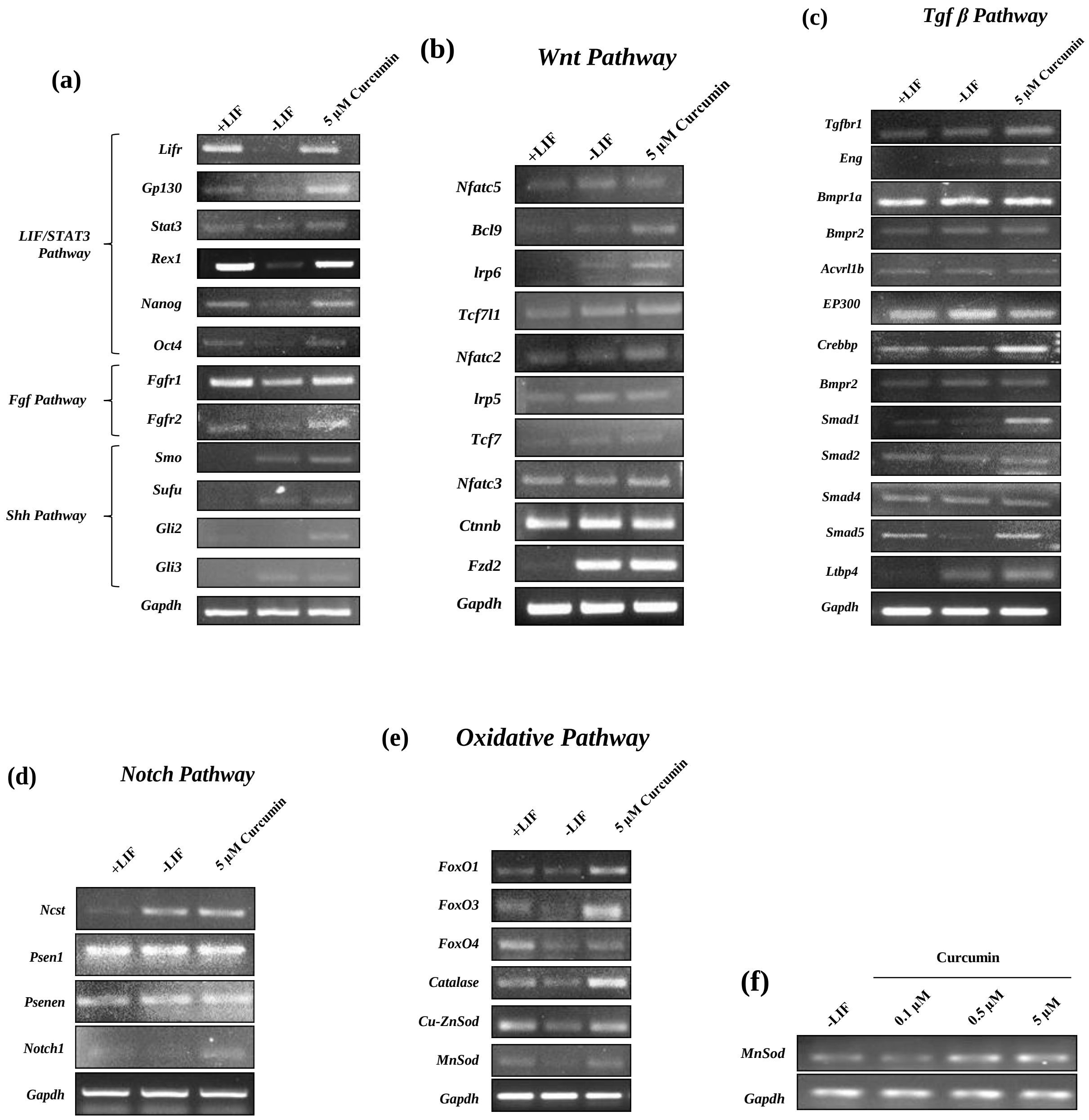

Supplementary Figure 4: Curcumin treated cells show enhanced expression of genes involved in LIF/STAT3 and Oxidative pathways**:** mESCs were cultured till confluency in the presence and absence of LIF and in the presence of 5 µM Curcumin and were harvested and analysed for the expression of various genes involved in pluripotency of mESCs – including LIF/STAT3, FGF, SHH (a) ,Wnt (b) Notch (c) TGFβ (d) and Oxidative (e) pathways by RT-PCR. f) *MnSod* expression analysis in mESCs treated with different concentrations of curcumin in the absence of LIF.

(a)

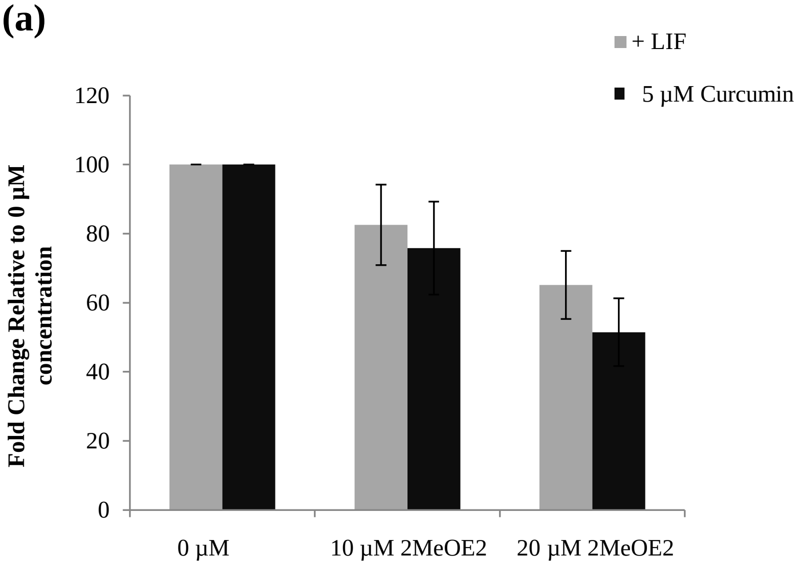

(b)

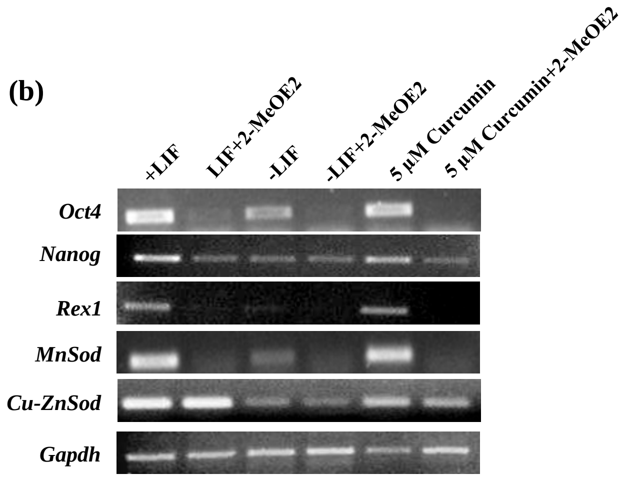

Supplementary Figure 5: 2-MeOE2 specifically acts on MnSOD and reduces SOD activity. mESCs were cultured under feeder free conditions in the presence and absence of 20 µM of MnSOD inhibitor2-MeOE2. 48 hrs post treatment, the cells were harvested for SOD activity (a) and Gene expression analysis (b). Graph shown is a representative of 3 experiments, depicted as mean ± S.E.M. 2-MeOE2 – 2-Methoxyestradiol.

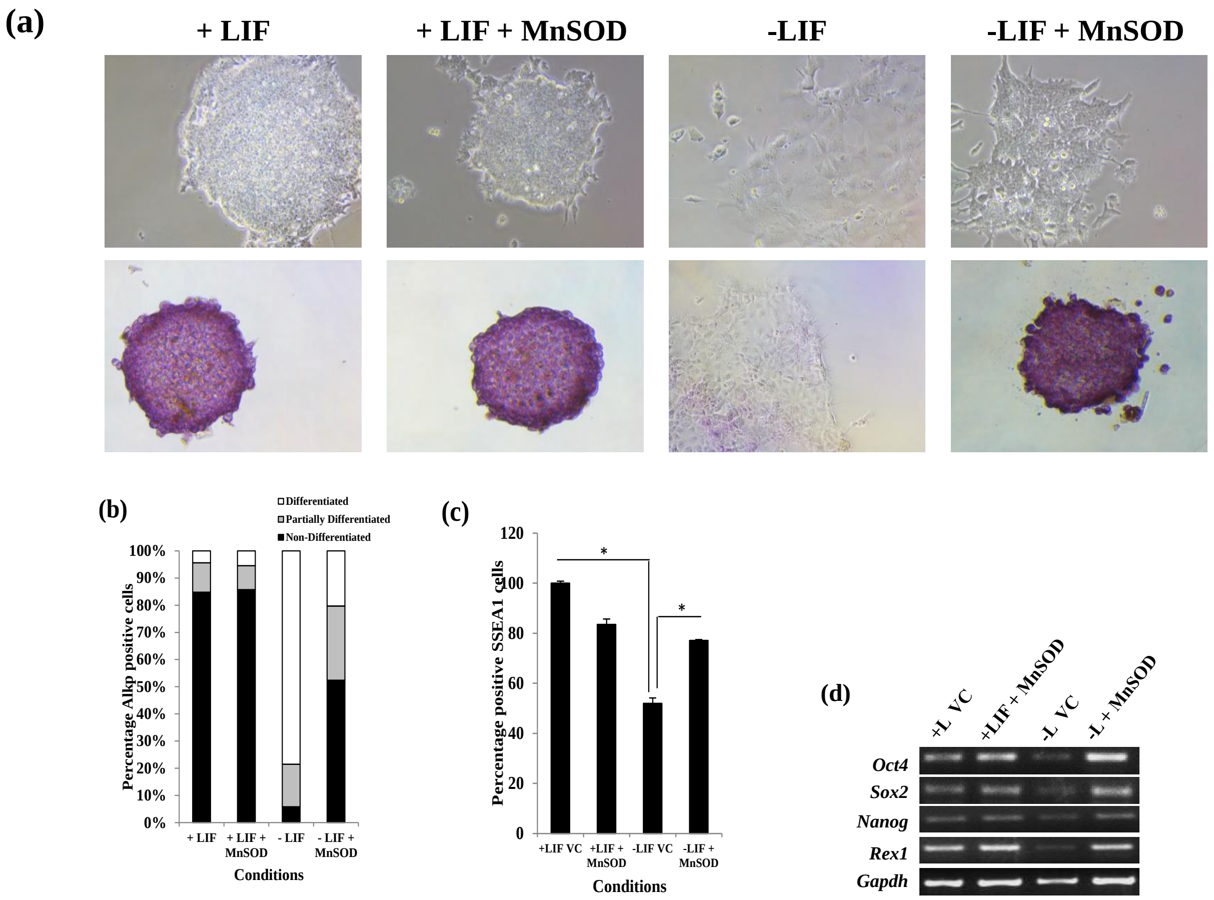

Supplementary Figure 6: MnSOD overexpression is sufficient to maintain pluripotency of mESCs. 20X magnified Phase Contrast Images and Alkaline Phosphatase stained images (a) of MnSOD overexpressed cells. MnSOD overexpressed cells were cultured on gelatin coated plates for 2 passages and at 3rd passage, the cells were used for Alkaline Phosphatase staining and Immunofluorescence. Approximately 10 cells were plated in each well of 12-well plate till day 6. On day 6, Alkaline phosphatase staining was performed and the number of alkaline phosphatase positive colonies were enumerated (b). Graph represents mean of 3 sets of experiments. (c) MnSOD overexpressed mESCs were cultured under feeder-free conditions and evaluated for SSEA1 surface marker expression by flow cytometry and the percentage positive cells, normalized to the "+LIF Vector Control" is plotted. Graph represents mean of 3 experiments ± S.E.M. (d) Pluripotent gene expression analysis of MnSOD overexpressed mESCs in the presence and absence of LIF and cultured till passage 10 by RT-PCR.

Supplementary Table 1: Sequence specific primers for the genes analyzed in this study

Sl No.	Gene Name	Gene Sequence	Amplicon Size
1	*Gapdh*	F: ACCACAGTCCATGCCATCAC	452
		R: TCCACCACCCTGTTGCTGTA	
2	*Oct4*	F: CTGCTGAAGCAGAAGAGGATCAC	399
		R: CTTCTGGCGCCGGTTACAGAACCA	
3	*Sox2*	F: AACATGATGGAGACGGAGCTGAAGC	500
		R: CTCCAGCCGTTCATGTGCGCGTA	
4	*Nanog*	F: TGAGCTATAAGCAGGTTAAGAC	139
		R: CAATGGATGCTGGGATACTC	
5	*Rex1*	F: CCAACAGGAAAGTGAATGGTG	195
		R: CAAAGTTGGCCATTCTTTAGGA	
6	*Gapdh Real Time*	F: AACTTTGGCATTGTGGAAGG	132
		R: GGATGCAGGGATGATGTTCT	
7	*Oct4 (Real Time)*	F: GAGGAGTCCAGGACATGAA	153
		R: AGATGGTGGTCTGGCTGAAC	
8	*Sox2 (Real Time)*	F: AAGAGATACAAGAGAATTGGGAGG	138
		R: AAGCGTTAATTTGGATGGGA	
9	*Foxg1*	F: ACCTGTCCCTCAACAAGTGC	300
		R: ACGTGGTCCCGTTGTAACTC	
10	*Pax6*	F: CGGCAGAAGATCGTAGAG	290/332
		R: GATGACACACTGGGTATG	
11	*βIIItubulin*	F: TAGACCCCAGCGGCAACTAT	127
		R: GTTCCAGGTTCCAAGTCCACC	
12	*Mixl1*	F: ACGCAGTGCTTTCCAAACC	196
		R: CCCGCAAGTGGATGTCTGG	
13	*Crypto*	F: CACTGGTTCCGCAAAGGACTA	102
		R: CCACGGGTCGAGGATGTAGA	
14	*Brachury*	F: TCCCGAGACCCAGTTCATAG	106
		R: TTCTTTGGCATCAAGGAAGG	
15	*Flk1*	F: CCCGCATGAAATTGAGCTAT	175
		R: AAACATCTTCGCCACAGTCC	
16	*Gata6*	F: CAACACAGTCCCCGTTCTTT	122
		R: TGGTACAGGCGTCAAGAGTG	
17	*Sox17*	F: GATGCGGGATACGCCAGTG	136
		R: CCACCACCTCGCCTTTCAC	
18	*Cxcr4*	F: CTTCTGGGCAGTTGATGCCAT	156
		R: CTGTTGGTGGCGTGGACAAT	

19	*FoxO1*	F: AAGAGCGTGCCCTACTTCAA	157
		R: CTCCCTCTGGATTGAGCATC	
20	*FoxO3*	F: ACAAACGGCTCACTTTGTCC	130
		R: CTGTGCAGGGACAGGTTGT	
21	*FoxO4*	F: GGTGCCCTACTTCAAGGACA	143
		R: GGTTCAGCATCCACCAAGAG	
22	*Cu-ZnSod*	F: CGGTGAACCAGTTGTGTTGT	190
		R: AGTCACATTGCCCAGGTCTC	
23	*MnSod*	F: CCGAGGAGAAGTACCACGAG	174
		R: GCTTGATAGCCTCCAGCAAC	
24	*Catalase*	F: TTGACAGAGAGCGGATTCCT	284
		R: GGCATCCCTGATGAAGAAAA	
25	*Trp53*	F: GGCCCCTGTCATCTTTTGT	120
		R: TGAGGGGAGGAGAGTACGTG	
26	*CyclinD1*	F: TGGTGAACAAGCTCAAGTGG	248
		R: GCAGGAGAGGAAGTTGTTGG	
27	*CyclinE1*	F: ACTTTCTGCAGCGTCATCCT	148
		R: TGTTGTGATGCCATGTAACG	
28	*Cdkn1b(p27kip1)*	F: GTGGACCAAATGCCTGACTC	122
		R: TCTGTTCTGTTGGCCCTTTT	
29	*Cdkn1a(p21cip1)*	F: TTCAGAGGCCGTTGTCTCTT	184
		R: TCTCCGTGACGAAGTCAAAG	
30	*Gp130*	F: CATGCTTTCAGGCTTTCCTC	242
		R: TCTCTGCTTCCACCCAGACT	
31	*Lifr*	F: GGTCAGCCCTGGAACTGTTA	266
		R: GAGCAGACCCTCTGTCGTTC	
32	*Stat3*	F: CCAGCAATATAGCCGATTCC	253
		R: CTGAAGATGCTGCTCCAACA	
33	*Fgfr1*	F: ATCCTCGGAAGATGATGACG	294
		R: GAAGGCACCACAGAATCCAT	
34	*Fgfr2*	F: CACCGAGAAGATGGAGAAGC	202
		R: GTCTGACGGGACCACACTTT	
35	*Smo*	F: CTACGGAGCCACCACCAC	168
		R: TCCACTCGGTCATTCTCACA	
36	*Gli2*	F: GGTCAAGACTGAGGCTGAGG	184
		R: CAGCTGCTCCTGTGTGTCAT	
37	*Gli3*	F: GCTCTTCAGCAAGTGGTTCC	125
		R: TTGCTGTCGGCTTAGGATCT	
38	*Sufu*	F: GGAACATGGGGAGTCCTTCT	126
		R: CAAATCCACTTGGTCCGTCT	

39	*Ncstn*	F: ATTGTGTGGGGGAAACTCAG	291
		R: GCTAGAGTCACGGCAAGACC	
40	*Notch1*	F: GAGATGCTCCCAGCCAAGT	143
		R: TCTTACACGGTGTGCTGAGG	
41	*Psen1*	F: CTTCCAGAATGCCCAGATGT	120
		R: ATTGGCTCAGGGTTGTCAAG	
42	*Psenen*	F: ATCTTGGTGGATTTGCGTTC	181
		R: TGGAAGATGGTGATCCAGGT	
43	*Crebbp*	F: CGCGAATGACAACACAGATT	120
		R: GCAGCATCTGGAACAAGGTT	
44	*Ep300*	F: CAAGCGGCCTAAACTCTCAT	142
		R: ATCGCCACCATTGGTTAGTC	
45	*Smad5*	F: AGATGGCCCCAGATAATTCC	248
		R: CAACAATCCCAGGCAGAATC	
46	*Smad1*	F: GCTGTGAGTTCCCATTTGGT	134
		R: AAGGCTGTGCTGAGGATTGT	
47	*Smad2*	F: AGGTGGTGGAGAGCAGAATG	176
		R: GTCCCCAAATTTCAGAGCAA	
48	*Smad4*	F: TCGATTCAAACCATCCAACA	199
		R: GCCCTGAAGCTATCTGCAAC	
49	*Avcr1b*	F: CGCTCCAGGATCTCGTCTAC	269
		R: AGCAGCAATAAAGCCAAGGA	
50	*Avcr1c*	F: TTCCAACAACGTGACCAAAA	261
		R: CCTGCATTCACAAGGCTGTA	
51	*Bmrp1a*	F: GGACCAGAAGAAGCCAGAAA	230
		R: CTTTCGGTGAATCCTTGCAT	
52	*Eng*	F: GACTCTTCAGGCATCCAAGC	258
		R: GGGTCATCCAGTGCTGCTAT	
53	*Tgfbr1*	F: AGAAGAGCGTTCATGGTTCC	243
		R: ATGTGAAGATGGGCAAGACC	
54	*Lrp5*	F: ACTGGACAGACTGGCAGACC	219
		R: GGCAGGCACAGGAGTAGAAA	
55	*Lrp6*	F: GGCAGCCAAATGCTACAAAT	130
		R: TTTCCATTCTCCAGCAGCTT	
56	*Bcl9*	F: GGGAAAAGGGAGCGAAGTAT	158
		R: GTAGCAGTTGACGGGGTCAT	
57	*Ctnnb1*	F: GGTGGACTGCAGAAAATGGT	222
		R: GCAGACAGACAGCACCTTCA	
58	*Nfat5*	F: GAGGGGAAGGAGCTGAAGAT	152
		R: TGCAACACCACTGGTTCATT	

59	*Nfatc3*	F: ATCGCCAGCACAAAAGTTCT	177
		R: TGGGATGTGCACACGAAATA	
60	*Tcf7*	F: CCCAGTCCTCCTCTCTACCC	129
		R: TACACCAGATCCCAGCATCA	
61	*Tcf7l2*	F: ATCGTCACACCGACAGTCAA	133
		R: TGAATGCATTAAGGGGCTTC	
62	*Sod2 Promoter*	F: CCCGGTACCGAAGACCGCTTTGACATCAGCCAT	1779
		R: CCCCTCGAGATTGACGTTTACACGACCGCTGCT	
63	*Sod2 gene Cloning Sequence*	F: CCCGGATCCCACCATGTTGTGTCGGGCGGCG	669
		R: CCCCTCGAGTCACTTCTTGCAAGCTGTGTATC	
64	*mShp2/ Ptpn11*	F:AGCTCTACTCCAGGGAAACAC	
		R:AATGAAGGTTCCTGTCCGGC	199
65	*mSocs3*	F:CCCAGGTCCTTTGCCTGATT	
		R:CGGGGAGCTAGTCCCGAA	215

Supplementary Table 2: Details of Antibodies used for Immunofluorescence and Western Blotting Procedures.

Sl. No.	Antibody Name	Company	Catalogue Number	Dilution	Technique used
1	Anti-NANOG	BD Biosciences, Torreyana Road, San Diego, CA. http://www.bdbiosciences.com/	560260	1:1000	ImmunoFluorescence
2	Anti-SSEA1	BD Biosciences, Torreyana Road, San Diego, CA. http://www.bdbiosciences.com/	557895	1:1000<br>1:100	ImmunoFluorescence<br>Flow Cytometry
3	Anti-OCT3/4A	BD Biosciences, Torreyana Road, San Diego, CA. http://www.bdbiosciences.com/	561555	1:1200	ImmunoFluorescence and Western Blotting
4	GAPDH	Santa Cruz Biotechnology, Inc. Dallas, Texas, U.S.A. http://www.scbt.com/	SC20357	1:750	Western Blotting
5	STAT3	BD Biosciences, Torreyana Road, San Diego, CA. http://www.bdbiosciences.com/	610189	1:2500	Western Blotting
6	pSTAT3	Cell signaling Technology, 3 Trask lane, Danvers, MA 01923, USA.http://www.cellsignal.com/	9131	1:1000	Western Blotting
7	MnSOD	Millipore, Single Oak Drive, Temecula, CA. http://www.emdmillipore.com/	06-984	1:1000<br>1:2000	ImmunoFluorescence<br>Western Blotting
8	NANOG	Millipore, Single Oak Drive, Temecula, CA. http://www.emdmillipore.com/	AB9220	1:1200	Western Blotting
9	β-TUBULIN	BD Biosciences, Torreyana Road, San Diego, CA. http://www.bdbiosciences.com/	556321	1:1200	Western Blotting
10	antimouse-IgM-AlexaFluor 488	BD Biosciences, Torreyana Road, San Diego, CA. http://www.bdbiosciences.com/	562409	1:2000	ImmunoFluorescence

11	antimouse IgG-PE	BD Biosciences, Torreyana Road, San Diego, CA. http://www.bdbiosciences.com/	349073	1:2000<br>1:500	ImmunoFluorescence<br>Flow Cytometry
12	Anti-Goat HRP	R&D systems, Minneapolis, MN, USA. http://www.rndsystems.com/	HAF109	1:2000	Western Blotting
13	Anti-mouse HRP	GE Healthcare LifeSciences, Buckinghamshire, HP7 9NA, UK. http://www.gelifesciences.com/	NA931V	1:2000	Western Blotting
14	Anti-rabbit HRP	GE Healthcare LifeSciences, Buckinghamshire, HP7 9NA, UK. http://www.gelifesciences.com/	NA934V	1:2000	Western Blotting
15	Anti-rabbit FITC	Millipore, Single Oak Drive, Temecula, CA. http://www.emdmillipore.com/	AP307F	1:250	ImmunoFluorescence

Supplementary Material and Methods:

Cell Culture

For experimental conditions, mESCs were cultured on both feeder and gelatin coated feeder-free conditions, in presence or absence of LIF conditions at the rate of 10,000 cells /well of six well plates in 2 mL medium. miPSCs were cultured only on feeder cells. After 24 hrs, the culture medium was replaced with mES medium lacking LIF, and curcumin dissolved in DMSO was added at various concentrations, while maintaining control cultures with the vehicle control (DMSO). Media change was given every 48 hrs to the cells and on confluency, the cells were harvested for gene and protein expression analysis.

For clonal analysis, mESCs were plated on gelatin coated 12 well plates with 10 cells/well in 1 mL medium. The cells were cultured in mES medium in presence or absence of LIF, and in presence of 5 µM Curcumin in no LIF condition. A single cell in each condition was identified, marked and traced for a period of 6 days to look for colony formation.

For Embryoid Body culture, the mESCs were cultured in low adherent plates (Corning) in the absence of LIF, with a media change every 3 days. The cells were harvested for gene expression analysis after 14 days of culture.

For Alkaline Phosphatase staining, mESCs cultured in presence and absence of curcumin were washed with PBS, fixed and stained with alkaline phosphatase substrate as per manufacturer's instruction (Millipore). After incubation with substrate for 15 min, colonies were stained purple and the image was captured using a phase contrast microscope (Nikon Eclipse TE2000S, Japan) and the number of alkaline phosphatase positive and negative colonies were enumerated.

Population Doubling

80,000 cells were cultured on feeder cells under LIF and No LIF conditions and in the presence of 5µM Curcumin. On the fourth day, the cells were trypsinized and 80,000 cells were replated on feeder cells while maintaining the said conditions through 8 passages. The cumulative population doubling was calculated and a graph was plotted as the number of cells/mL vs. the number of days in culture.

Gene Expression Analysis

RNA isolation was performed using RNA micro kit (Qiagen) as per manufacturer's instruction. cDNA was prepared using 1 µg of RNA according to the manufacturer's instructions (SuperScript III First Strand cDNA Synthesis kit, Invitrogen). Conventional Polymerase Chain Reaction (PCR) was performed using 2X Master Mix (Fermentas) and 5µM gene specific primers (IDT technologies). Quantitative PCR was carried out using SYBR Green (Kapa Biosystems). The samples were analysed using the 7500 RT-PCR (ABI Biosystems) and were normalized with *Gapdh* to obtain the relative Δ Ct values among samples.

Immunofluorescence

Cultured ESCs in the presence and absence of LIF and medium containing 5 µM curcumin or MnSOD overexpressed or MnSOD ShRNA cells were fixed in 4 % paraformaldehyde at 4°C for 20 mins and then permeabilized with 0.2 % Triton X-100 in PBS for 30 minutes, and blocked in blocking buffer containing 3 % BSA containing 10 % Donkey serum in 1X PBS Solution and incubated at room temperature (RT)

for 30 mins. Antibodies were diluted in blocking buffer and cells were incubated in the indicated antibody solution at 4 °C overnight, followed by incubation for 2 hrs at RT with Secondary antibody. The cells are washed with 0.1 % PBS-Tween 20 and stained with DAPI (Sigma Aldrich) for 1 minute and observed under fluorescent microscope (Nikon Eclipse TE2000U, Japan). Both Purified and conjugated antibodies were used. The primary antibodies and dilutions used are mentioned in the supplementary table 2.

Cloning

The Open Reading Frame (ORF) of MnSOD was PCR amplified from mESC cDNA using gene specific primers as mentioned in supplementary table 1. The PCR product corresponding to the complete size of MnSOD was cloned in between BamHI and XhoI sites of pCDNA3.1 (+) (for transient transfection) and pMIG vector (for retroviral transfection) and transformed into competent DH5α cells. The ampicillin resistant colonies were selected and screened for positive clones and confirmed by sequencing (Amnion Biosciences, Bangalore).

mESC genomic DNA was isolated using genomic DNA isolation kit according to manufacturer's protocol (Genetix Biotech). As reported earlier, 1779 bp upstream region from the start site of MnSOD was identified, PCR amplified and cloned into KpnI and XhoI regions of pGL3 basic vector (Addgene).

Transfection

For transient transfection, 5 µg of plasmid [MnSOD and vector control pCDNA 3.1(+)] was transfected into confluent mESCs using Xtremegene HP transfection reagent according to manufacturer's instructions (Roche diagnostics). 24 hrs post transfection, the transfected cells were subjected to different conditions (With LIF, No LIF) and cultured till confluency. The cells were then passaged. On confluency, the cells were analysed for their gene and protein expression pattern.

pMIG-MnSOD and pMIG-GFP were transduced into mESCs using retroviral particles. Briefly, pMIG-MnSOD and pMIG-GFP plasmids were packaged into retroviral packaging vectors pVSVG and pGAGPOL in 293T cells. Retroviral transduction was performed on 80% confluent mESCs twice in 24 hr intervals. 48 hrs post-transduction, the mESCs were trypsinised using 0.25% trypsin and plated on inactivated MEF and cultured in the presence and absence of LIF. Media change was given every 48 hrs to the cells and on confluency, the cells were passaged and at P10, the cells were harvested for gene expression analysis.

MnSOD and Cu-Zn SOD ShRNA were transduced into mESCs using lentiviral particles. Briefly, pLKO-MnSODshRNA and pLKO-Cu-ZnSODshRNA (Sigma Aldrich, Germany) along with the scrambled control, were each packaged into lentiviral packaging plasmids – psPAS2 and pMD.2G, along with the scrambled control. Lentiviral transduction was performed on 80 % mESCs twice in 24 hr intervals. 24 hrs post 2nd transduction, the transduced mESCs were trypsinised using 0.25 % trypsin and plated on gelatine coated 6 well plate. The cells were then selected with 1µg of puromycin, with media change every 48 hrs. On reaching 80 % confluency in the second passage, the cells were harvested for gene and protein expression analysis.

Western Blotting:

Cells were harvested and were lysed with ice-cold RIPA cell lysis buffer with freshly added protease inhibitors and phosphatase inhibitor. Protein concentration was determined using BCA kit (Novagen). 20-

50 µg of protein was used for western blotting experiments. The protein were run on 8-12 % SDS-PAGE gels and transferred onto charged PVDF membrane (Millipore). Post transfer, the membrane was blocked using 3 % Skimmed milk/BSA in 0.1% TBST solution and probed with primary antibody overnight at 4 ºC and subsequently with horseradish-peroxidase (HRP)-conjugated secondary antibody for 1 hr at RT. The protein specific bands were detected by chemiluminiscence analyser (GE ImageQuant LAS 4000) or using a TMB substrate (Sigma Aldrich). Details of the antibodies used are mentioned in the supplementary table 2.

Teratoma Assay

To evaluate the pluripotency of curcumin treated cells, approximately 1-3 $*10^6$ ES cells cultured in absence of LIF and in presence of curcumin were harvested and injected subcutaneously (s.c.) into the dorsal flank of 4-5 week old female athymic nude mice. Following 4 weeks, mice were sacrificed and the tissues were removed, fixed with formalin and embedded with paraffin. Further sectioning and H&E staining was performed by Anand Diagnostics, Bangalore. The tissue sections were then observed under the microscope for the identification of different lineage cell types using a phase contrast microscope (Nikon Eclipse TE2000S, Japan).

Flow cytometric analysis

Oct4-GFP iPSCs were cultured for 4-5 days in absence of LIF and in presence or absence of Curcumin and were trypsinized and subjected to flow cytometric analysis to enumerate the number of GFP expressing cells using a flow cytometer (BD FACS Calibur, U.S.A).

For Propidium iodide staining, confluent mESCs treated in the presence and absence of LIF and in the presence of curcumin were stained with propidium iodide (Sigma Aldrich) for cell cycle analysis by flow cytometry.

MnSOD overexpressed cells were analysed for SSEA1 expressing population by Flow cytometry. Briefly, MnSOD overexpressed cells were trypsinised and washed with 1X PBS. The cells were then stained with Anti-SSEA1 antibody, for 1 hr on ice. The cells were then washed in PBS and stained with PE conjugated anti-mouse IgG antibody for an hour on ice. The cells were then fixed with 4% PFA for 1 hr on ice and then analysed flow cytometrically. The number of positive SSEA1 cells were then selected by gating out the secondary alone and cells alone controls and plotted.

BrDU based Cell Proliferation Assay

700-1000 cells were plated in each well of a 96 well plate. 24 hrs post plating, 0.2 µg/mL of BrDU was added to the proliferating cells and incubated for 12-16 hrs at 37 ºC, 5 % CO_2. Cells were then fixed with ice cold 70 % ethanol for 10 mins at RT. The cells were permeabilised with 0.1 % Triton X-100 in PBS for 1 hr at RT and the BrDU labelled nuclei were detected using anti-BrDU antibody (1:1000 dilution; Sigma Aldrich, B8434) and secondary anti-mouse HRP conjugated antibody (1:2000 dilution; GE, Catalogue number NA931V) for 1 hr at RT and developed using TMB solution. The reaction was stopped using 3M H_2SO_4 and the absorbance was read at 450 nm.
